# Genome-wide analysis of AP2/ERF transcription factors that regulate fruit development of Chinese prickly ash

**DOI:** 10.1186/s12870-024-05244-9

**Published:** 2024-06-15

**Authors:** Lei Ma, Qianqian Shi, Qin Ma, Xiaona Wang, Xin Chen, Peilin Han, Yingli Luo, Haichao Hu, Xitong Fei, Anzhi Wei

**Affiliations:** 1 College of Forestry, Northwest Agriculture and Forestry University, Yangling, Xianyang, 712100 China; 2 Research Centre for Engineering and Technology of Zanthoxylum State Forestry Administration, Yangling, Xianyang, 712100 China

**Keywords:** AP2/ERF transcription factor, Expression pattern, RT-qPCR, Candidate gene, Fruit development, Chinese prickly ash

## Abstract

**Background:**

AP2/ERF is a large family of plant transcription factor proteins that play essential roles in signal transduction, plant growth and development, and responses to various stresses. The AP2/ERF family has been identified and verified by functional analysis in various plants, but so far there has been no comprehensive study of these factors in Chinese prickly ash. Phylogenetic, motif, and functional analyses combined with transcriptome analysis of Chinese prickly ash fruits at different developmental stages (30, 60, and 90 days after anthesis) were conducted in this study.

**Results:**

The analysis identified 146 ZbAP2/ERF genes that could be classified into 15 subgroups. The motif analysis revealed the presence of different motifs or elements in each group that may explain the functional differences between the groups. *ZbERF13.2*, *ZbRAP2-12*, and *ZbERF2.1* showed high levels of expression in the early stages of fruit development. *ZbRAP2-4*, and *ZbERF3.1* were significantly expressed at the fruit coloring stage (R2 and G2). *ZbERF16* were significantly expressed at fruit ripening and expression level increased as the fruit continued to develop. Relative gene expression levels of 6 representative ZbAP2/ERFs assessed by RT-qPCR agreed with transcriptome analysis results.

**Conclusions:**

These genes identified by screening can be used as candidate genes that affect fruit development. The results of the analysis can help guide future genetic improvement of Chinese prickly ash and enrich our understanding of AP2/ERF transcription factors and their regulatory functions in plants.

**Supplementary Information:**

The online version contains supplementary material available at 10.1186/s12870-024-05244-9.

## Background

The Rutaceae family includes many important fruits tree species, such as grapefruit (*Citrus paradisi*), orange (*Citrus aurantium*), tangerine (*Citrus reticulata*), and Chinese prickly ash (*Zanthoxylum bungeanum*). The ripe fruit are utilized, so fruit quality is a key factor in determining the economic value. Fruit development directly affects the quality of the fruit at maturity, and understanding the genetic regulation of fruit development can be helpful in improving these plants. The AP2/ERF family of transcription factors (TF) family includes proteins that are ubiquitous in a variety of plants and with functions involved in plant growth and development, as well as in biotic and abiotic stress responses [1]. Understanding the basis for gene regulation by these transcription factors has great application value in crop genetic engineering and breeding. Transcriptional factors bind specifically to *cis*-acting elements in eukaryotic gene promoters to control gene expression [2]. Interactions with other proteins may further activate or inhibit gene expression to ensure that a target gene is expressed to a specific level at a particular time and location. All AP2/ERF members contain a DNA-binding AP2 domain, consisting of 60 to 70 amino acids [3]. According to the number and similarity of AP2 domains and the presence or absence of other domains, AP2/ERF transcription factors can be classified into five subfamilies: APETALA2 (AP2), Ethylene-responsive factor (ERF), Dehydration-responsive element binding protein (DREB), Related to ABI3/VP1 (RAV), and Soloist. Most proteins are in the ERF and CBF/DREB subfamilies [4]. ERF and DREB subfamily proteins contain one AP2 domain, the AP2 subfamily contains two repeated AP2 domains, and the RAV subfamily proteins contain an AP2 domain and a highly conserved B3 domain [1, 2]. The Soloist group contains one AP2 domain and forms a separate group because it is structurally distinct from other members of the AP2/ERF family. Despite the highly conserved sequence of the AP2 domain, each subfamily has a completely different DNA-binding element. The ERF subfamily is further subdivided into B1 ~ B6 subgroups, which can bind GCC-box (AGCGCCC) elements; the DREB subfamily can be divided into A1 ~ A6 subgroups, which can bind dehydration response elements/C-repeat and RCCGCC elements [4]. There are two tandemly repeated AP2 domains in the AP2 subfamily that do not bind to the GCC-box but to the GCAC(A/G)N(A/T)TCCC(A/G)ANG(C/T). As well, the RAV subfamily binds to elements such as CACCTG and CAACA [5, 6].

Several studies have identified and validated that AP2/ERF transcription factors can regulate different periods of plant development and different response patterns to stresses, such as reproductive development, cell proliferation, abiotic and biotic stress responses, and phytohormonal responses [1, 7]. The AP2 structural domain was reportedly first discovered and identified as a recurrent theme in the Arabidopsis AP2 protein, which is involved in flower development [8]. Genes in the AP2 subfamily have important roles in regulating organ structure, leaf development, embryonic development, and other organ architecture and development in plants [9]. The RAV subfamily of genes is involved in plant hormone signaling, including ethylene and auxin, and is a major regulator of various stress responses [10]. Stress responses and developmental stages are regulated by the ethylene signaling pathway [11]. Ethylene promotes the expression of specific transcription factors of the ERF subfamily in plants. In fruit ripening, ethylene is a critical factor, and AP2/ERF regulates ethylene metabolism [12, 13]. A significant association was found between fruit development and the expression levels of genes from the ERF family. ERF subfamily transcription factor *PS-ERF1* regulates plum fruit ripening via the ethylene pathway [13]. The banana AP2/ERF family gene *MaERF9* positively promotes the expression of the ethylene-related gene *MaACO1* and another gene, *MaERF11*, regulates ethylene-mediated banana fruit ripening by negatively regulating transcription from the promoters of *MaACO1* and *MaACS1* [14]. Some members of the ERF subfamily have been found to enhance abiotic stress tolerance in plants and exhibit different response patterns under abiotic stresses after characterization as candidate genes. The AP2/ERF superfamily has been genomically characterized in many species. 147 gene members were identified in Arabidopsis and 163 members were published in rice (*Oryza sativa*) [15]. There are 202 family members identified in poplar (*Populus trichocarpa*) [16]. Among the gymnosperms, 88 members are in Masson pine (*Pinus massoniana*) [17]. In fruit trees, there were 149 genes in grapes (*Vitis vinifera*) [7] and 126 members of AP2/ERF in citrus (*Citrus reticulata*) [18]. A total of 146 AP2/ERF family members were identified from green and red prickly ash, which were close to 147 and 149 in Arabidopsis and grape, respectively.

The cultivation of Chinese prickly ash dates back nearly 2000 years in China. It is widely distributed in Asia, and the medicinal value of prickly ash has long been recognized. Modern medical research has identified antioxidant, insecticide, anesthesia, anti-inflammatory, analgesic, lowering blood fat, wrinkle reduction, and anti-cancer properties [19–21]. Chinese prickly ash is an important economic tree, and is used widely as a condiment and spice due to its unique numbing taste and aroma, especially for the characteristic flavoring of hot pots [22]. In addition, the volatile oil extracted from the peel of the fruit has high antioxidant activity and excellent free radical scavenging ability, making it attractive for use in the cosmetic industry. Chinese prickly ash has a well-developed root system, is drought-resistant and tolerant of poor soil, and has excellent soil fixing ability, making it a good ecological species to plant on barren hills for urban greening and conservation projects. However, prickly ash is affected by site conditions and environmental factors during industrial development, resulting in reduced yield and stability and reducing the medicinal and flavoring qualities of prickly ash. The lowered quality reduces sales, thus causing economic losses and limiting the development of the industry. Therefore, it is necessary to improve the resistance and quality of Chinese prickly ash through genetic breeding-related research. Given the importance of AP2/ERF transcription factor family members is regulating fruit development in other plant species, we hypothesized these factors will also play important roles in Chinese prickly ash. However, little attention has been paid to the AP2/ERF in this species. In this work, we comprehensively analyzed the functions, patterns, structures, and predicted protein interaction networks of ZbAP2/ERF TFs and screened nine candidate genes by analyzing their expression patterns in different developmental stages of fruits. The results of the analysis can guide the molecular breeding research of Chinese prickly ash and also help us understand the functions of AP2/ERF superfamily in different plants.

## Results

### Identification of conserved domains and prediction of physicochemical properties of AP2/ERF proteins in Chinese prickly ash

A total of 146 genes containing AP2/ERF domains in Chinese prickly ash were identified in the Pfam database based on the AP2 domain number (PF00847). The sequences of ZbAP2/ERF proteins were named based on similarity to the AP2/ERF TF sequences of Arabidopsis and the Blast results of AP2/ERF TF sequences of other species (Supplementary Table 1). The predicted proteins ranged in length from 121 to 694 amino acids, with molecular weights between 13.405 and 76.253 kDa. Their theoretical isoelectric points (pIs) ranged from 4.29 to 9.98, with a mean value of 6.68, indicating that most ZbAP2/ERF proteins are weakly acidic (Supplementary Table 2).

### Phylogenetic analysis of AP2/ERF genes and classification

**Fig. 1:**
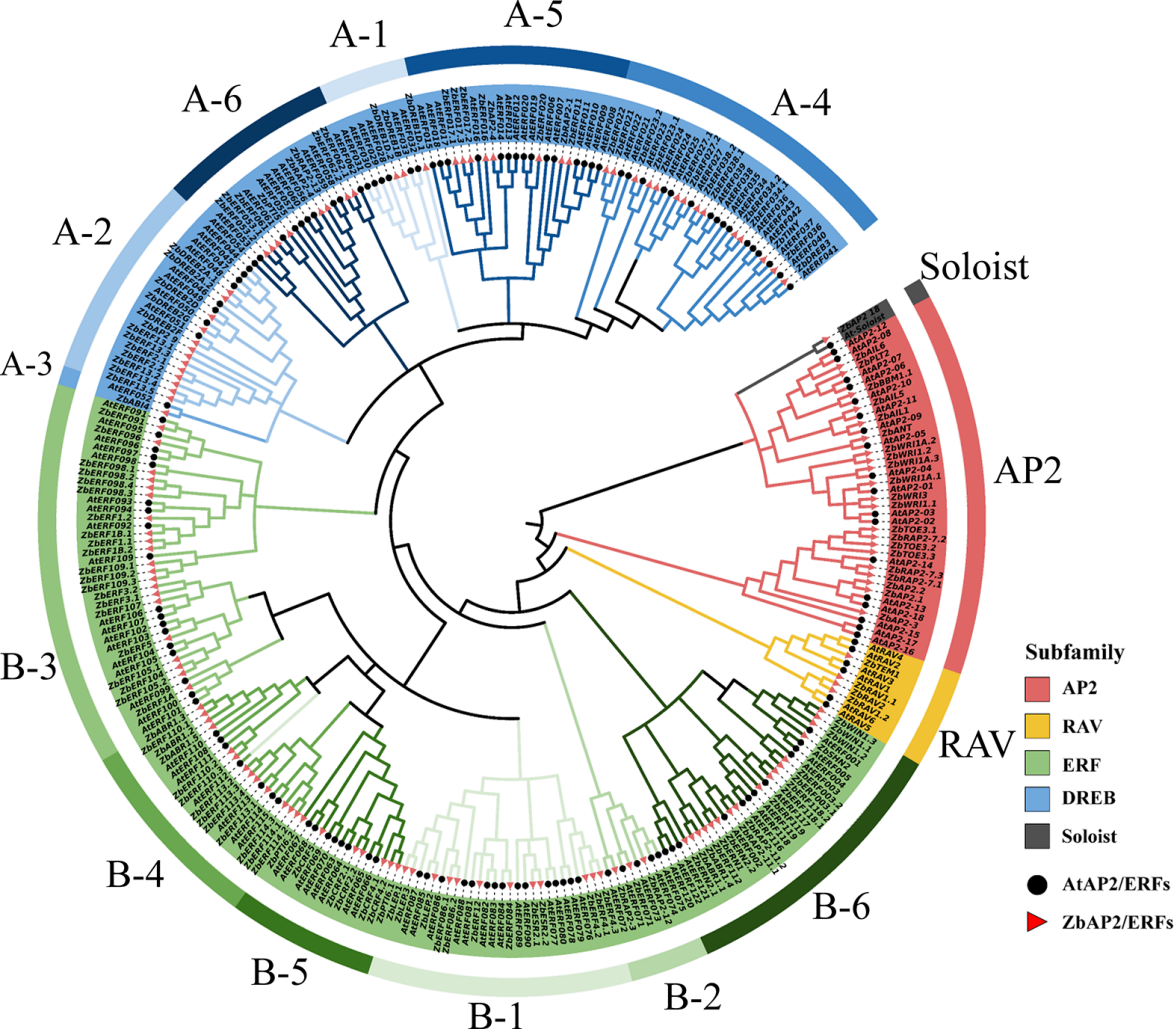
Phylogenetic tree of Chinese prickly ash and Arabidopsis AP2/ERF transcription factors. The inner circle indicates the classification of each subfamily in the phylogenetic tree, and the outer circle indicates the further classification of the ERF subfamily (B1-B6) and the further classification of the DREB subfamily (A1-A6)

The conserved domain features of AtAP2/ERF and ZbAP2/ERF proteins were analyzed using the ClustalW multiple sequence alignment tool and a phylogenetic tree was constructed consisting of 146 prickly ash AP2/ERF TFs and 146 Arabidopsis AP2/ERF TFs using MEGA-X software (Fig. 1). The results of multiple sequence alignment of Arabidopsis and Chinese prickly ash AP2/ERF family genes are shown in Supplemental Fig. 1. The grouping of ZbAP2/ERF factors was performed with reference to the grouping method of AP2/ERF in Arabidopsis [23]. Referring to the grouping method of Sakuma [4], the identified genes were divided into AP2, ERF, DREB, RAV, and Soloist groups. According to the classification results, the AP2 subfamily has 21 members, the DREB subfamily has 47 members, the ERF subfamily has 73 members, the RAV subfamily has four members, and one gene was classified into the Soloist group. There were fewer prickly ash genes classified into the RAV and DREB subfamilies than in Arabidopsis (6 and 56, respectively) but more prickly ash genes classified into AP2 and ERF subfamilies that in Arabidopsis (18 and 65, respectively). Prickly ash gene *ZbAP2_18* is similar to *At4g13040* (Nakano) and *At079349* (Sakuma) in Arabidopsis, contains an AP2 domain, but has low homology with other subfamily genes, so was designated as Soloist based on the previously used classification method for Arabidopsis thaliana.

### Analysis of the motifs and structural domains present in ZbAP2/ERF family genes

**Fig. 2:**
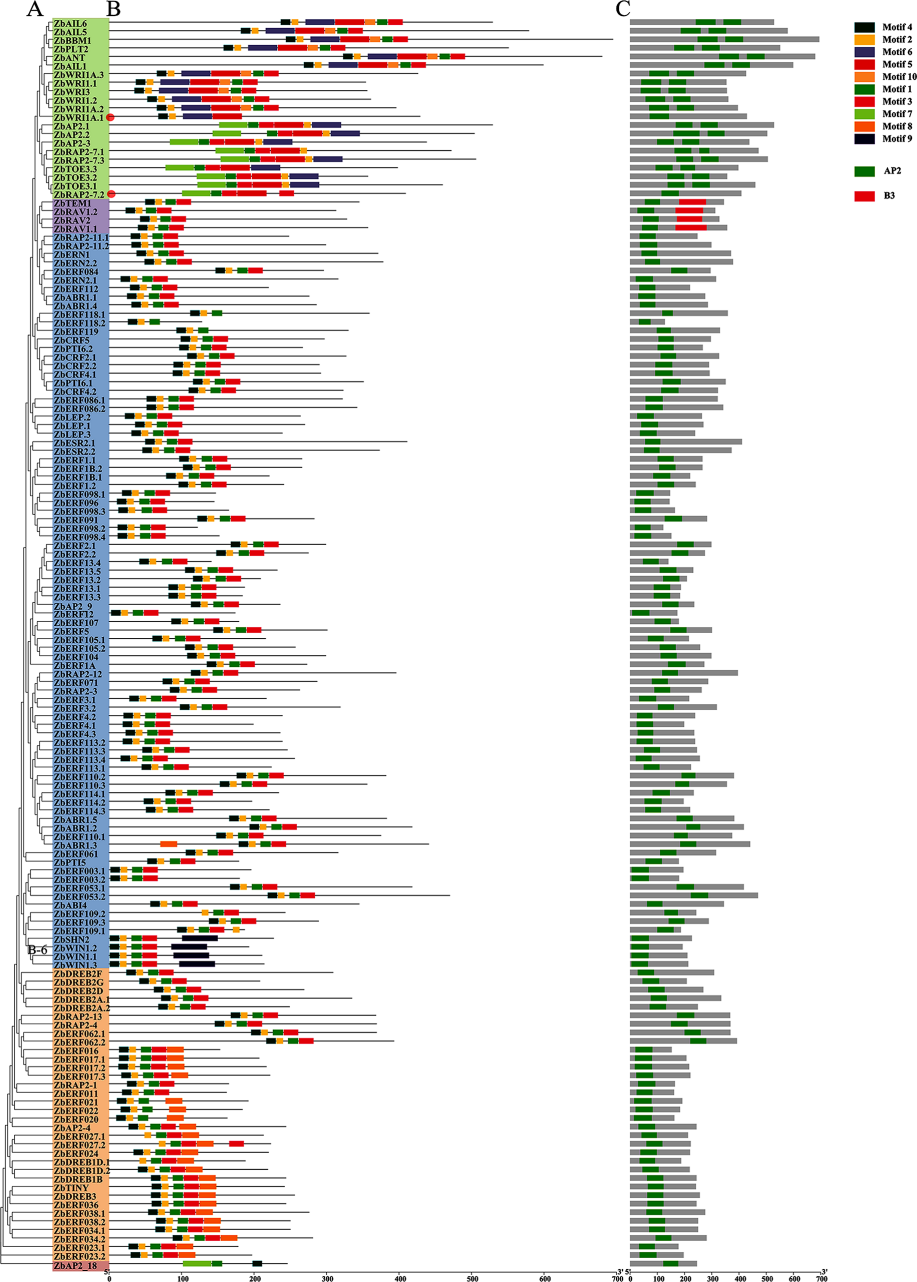
Motif discovery and structural domain visualization of ZbAP2/ERF family genes. (**A**) Phylogenetic tree classification of subfamilies (AP2 subfamily on green background, RAV subfamily on purple background, ERF subfamily on blue background, DREB subfamily on yellow background, Soloist subgroup on pink background); (**B**) Motif sites and types of the ZbAP2/ERF family; (**C**) Structural domain sites and types of the ZbAP2/ERF family

Motif discovery was performed for the ZbAP2/ERF family genes and the discovered motifs were limited to 10 (Fig. 2A-B), of which 10 motifs are shown in Supplementary Fig. 3. The results showed that Motif 1 and Motif 2 are common to all members of the family, suggesting that these motifs are essential components of the AP2 conserved structural domain (Fig. 2B). In terms of subfamilies, motifs 5 and 6 are unique to the AP2 subfamily; AP2 subfamily members *ZbWRI1A.1* and *ZbRAP2-7.2* contain only one AP2 structural domain but belong to the AP2 subfamily in terms of homology, and motif analysis shows that motif 5 is the only motif common to both, suggesting that motif 5 may be the key motif for identifying the AP2 subfamily. In the ERF subfamily there are common motifs 1, 2, 3 and 4 and some of the members contain a unique motif, motif 9, which can be recognized as a characteristic motif of the B-6 family members according to the phylogenetic tree. Motifs 1, 4 and 7 are present in the soloist group, but the major difference from all other members is that motif 4 is located near the 3’ end rather than the 5’ end, which may be the main reason for the lack of homology of *ZBAP2_18* with all other members. Each subfamily has some unique motifs, which are the characteristic sequences for clustering and differentiating the AP2/ERF superfamily, and are also responsible for the functional differences between the subfamilies. The domains of these sequences were searched in the NCBI-CDD database and visualized (Fig. 2C). Members of the ZbAP2 subfamily have two concatenated, repeating AP2 domains and an additional B3 domain in ZbRAV. The two members of the AP2 subfamily, *ZbWRI1A.1* and *ZbRAP2-7.2*, contain only one AP2 domain, but these two genes are highly homologous to other members of the ZbAP2 subfamily and are classified as the ZbAP2 subfamily on the phylogenetic tree.

### Comparative multiple sequence analysis of ZbAP2/ERF family genes

**Fig. 3:**
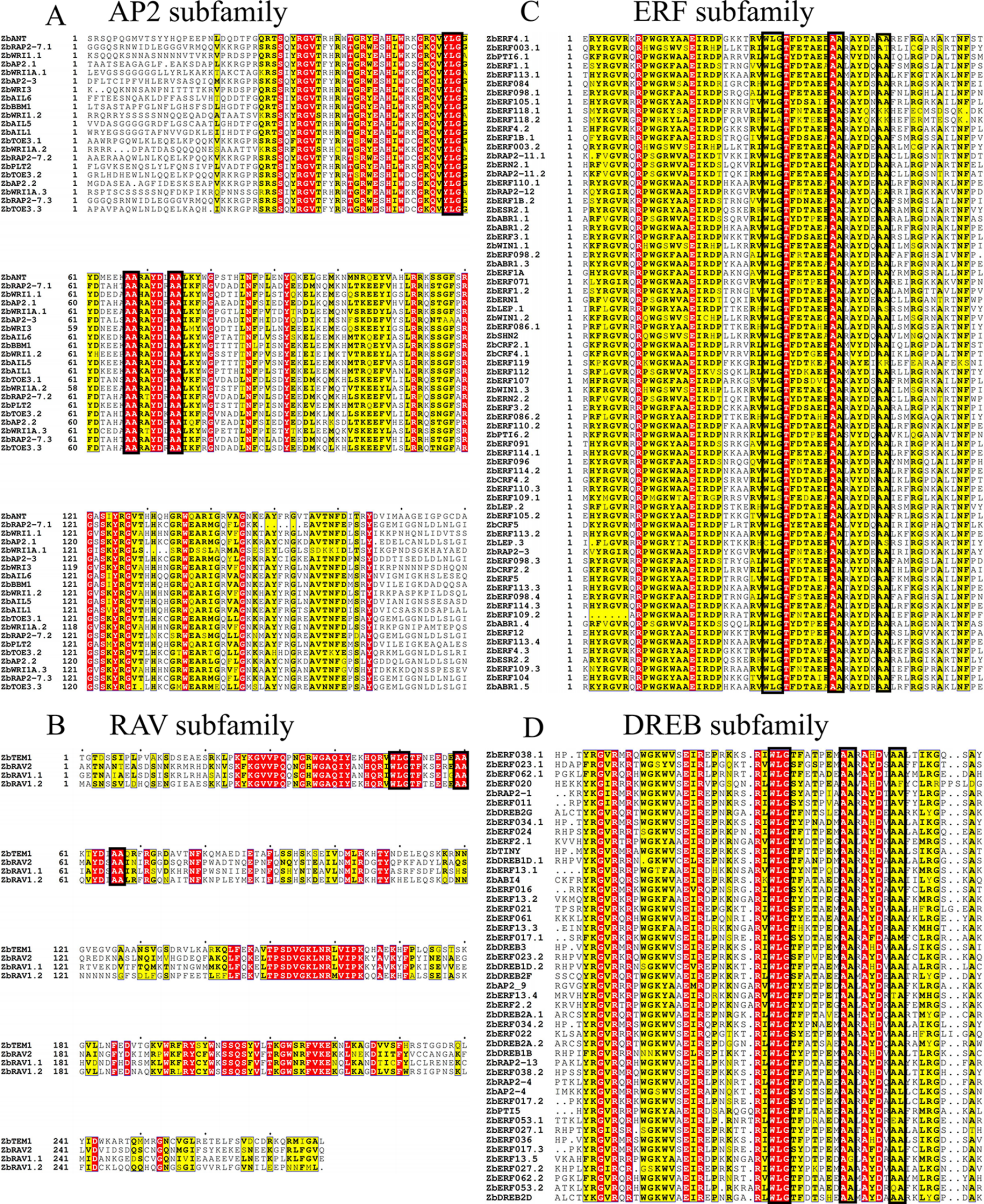
Multiple sequence alignment of the ZbAP2/ERF family. (**A**) Sequence alignment of the AP2 subfamily; (**B**) Sequence alignment of the RAV subfamily; (**C**) Sequence alignment of the ERF subfamily; (**D**) Sequence alignment of the DREB subfamily

All ZbAP2/ERF members exhibit two fully conserved elements, AA and WLG, as depicted in (Fig. 3A-D). The RAV subfamily stands out with the longest conserved range, featuring two conserved structural domains. Additionally, only genes of the AP2 subfamily display variation in the fully conserved WLG, with a transformation of WLG to YLG. Moreover, while two AA elements remain fully conserved across all members, certain members also demonstrate variation in highly conserved elements such as RAYD and YRG. For example, RAYD was mutated to RT/KTYD and YRG was mutated to YKG (Fig. 3A). Four genes from the ZbRAV family in the RAV subfamily were found to contain AP2 structural domains within the first 60 amino acids of the 120 amino acid length and B3 structural domains in the second half of the same 120 amino acid region. Additionally, they showed fully conserved elements of WLG and AA, which is consistent with other subfamilies (Fig. 3B). Similar to the ERF subfamily in other species, the ZbERF gene contains conserved elements of YRG, AEIR, WLG, AA, and RAYD. However, there are some variations in these elements, such as AEIR being mutated to SEIR and RAYD being mutated to L/HAYD (Fig. 3C). Thirteen residues in the DREB subfamily are completely preserved in all ZbDREBs (Fig. 3D). As with other plant DREB sequences, the WLG element is completely conserved in these sequences. Additionally, YRG, RAYD, and AA elements are highly conserved.

### Analysis of the expression patterns of ZbAP2/ERF genes in different developmental stages of fruits

**Fig. 4:**
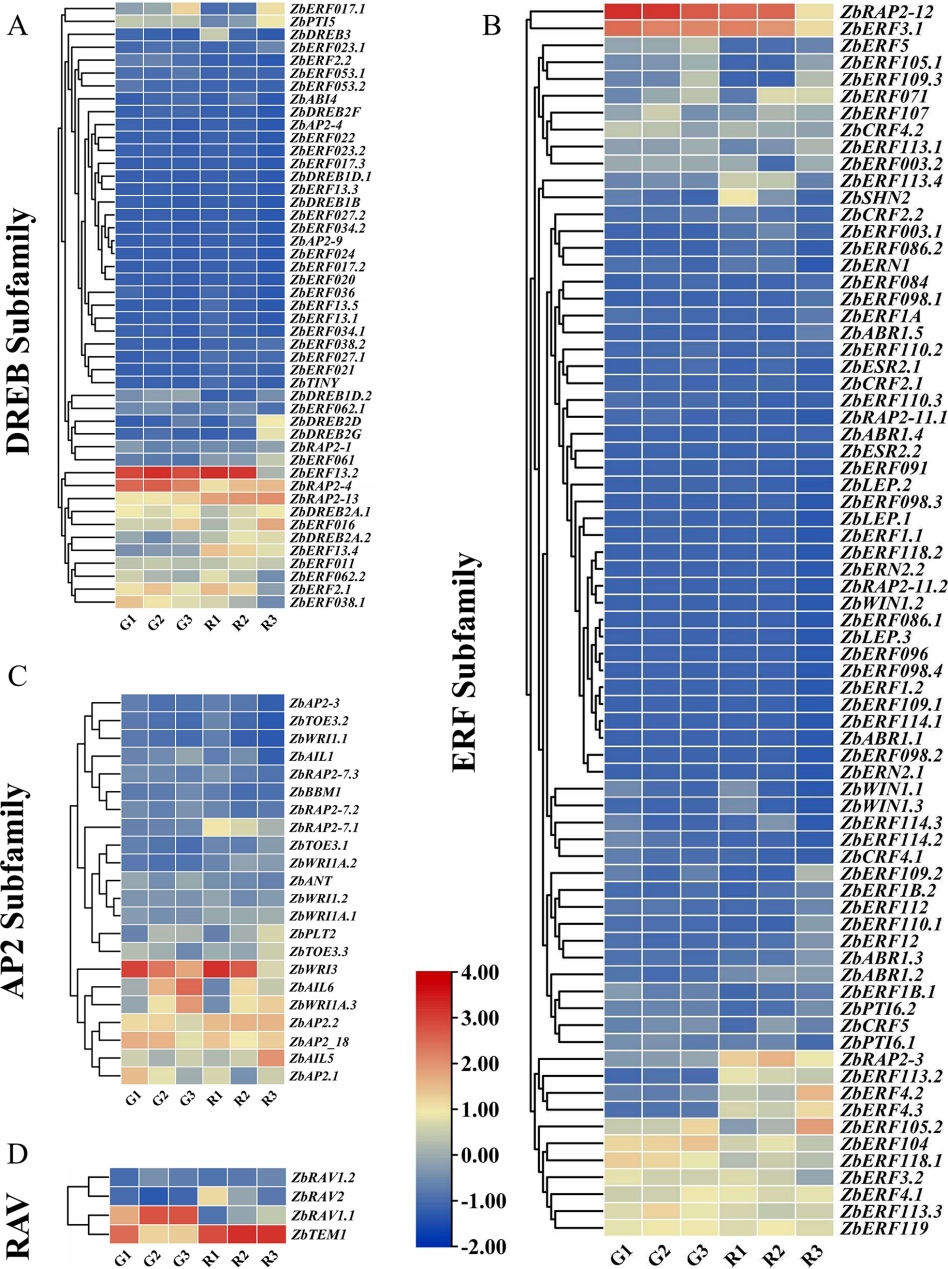
Expression profiles of 147 ZbAP2/ERF genes based on transcriptome data from different developmental stages of the fruit. Expression profiles of (**A**) DREB subfamily; (**B**) ERF subfamily; (**C**) AP2 subfamily; (**D**) RAV subfamily and Soloist genes; (in |log2fold change| values). Red (R) and green (G) fruit samples were collected at three different developmental stages: 30 days after flowering (R1 and G1), 60 days after flowering (R2 and G2), and 90 days after flowering (R3 and G3)

Using transcriptome data, the expression profiles of 146 ZbAP2/ERF genes in the unstressed pericarp of green prickly ash (G) and red prickly ash (R) were analyzed at three different developmental stages (30, 60, and 90 days after flowering) (Fig. 4). The results showed that some genes showed increasing expression levels during the continuous development of *Zanthoxylum bungeanum* fruit. *ZbERF13.2*, *ZbRAP2-12*, and *ZbERF2.1* showed high levels of expression in the early stages (R1 and G1) of fruit development. *ZbRAP2-4*, and *ZbERF3.1* were significantly expressed at the fruit coloring stage (R2 and G2) (Fig. 4A-B). *ZbERF16* were significantly expressed at fruit ripening and expression level increased as the fruit continued to develop (Fig. 4A). Overall, there was little difference in the expression patterns of ZbAP2/ERF TFs in green and red prickly ash, suggesting these genes are equally important for ripening in the two kinds of prickly ash. For example, *ZbERF017.1* and *ZbERF016* were both up-regulated, and *ERF038.1*, and *ZbWRI3* were both down-regulated in the same stages for green prickly ash and red prickly ash (Fig. 4A-C). For red prickly ash, there was high expression of most genes in the ripening stage of the fruit (R3), with increasing expression with fruit development. *ZbERF13.2*, *ZbERF13.4*, and *ZbERF2.1* exhibited high expression in the early stage of fruit development (R1), with *ZbERF13.2* showing the highest expression level, and then all three genes showed down-regulation in later stages. In green prickly ash, most genes exhibited the highest expression levels in the middle stage of fruit development (G2), with an overall trend of first increasing first and then decreasing with the development of fruit. The expression analysis suggests that these genes play essential roles in the development and maturity of green and red prickly ash fruits, and their activities may be closely related to growth and development and the biosynthesis of secondary metabolism. In addition, transcriptome data were analyzed for differential expression (Supplementary Fig. 1). Hence, a differential gene analysis (R3vsG3, R2vsG2) was conducted on all member genes of green and red prickly ash at the ripening and fruit coloring stages. The fold change (FC) in log2FC denotes the ratio of expression in green and red prickly ash during the ripening period, where the log2FC is calculated after taking the logarithm to the base of 2. Specifically, the ripening stage of green prickly ash exhibited 8 up-regulated genes and 16 down-regulated genes, while the coloring stage showed 5 up-regulated and 7 down-regulated genes. Green prickly ash displayed 8 up-regulated genes and 16 down-regulated genes at the ripening stage when compared with red prickly ash, and 5 up-regulated genes and 7 down-regulated genes at the coloring stage. Notably, *ZbERF13.2* was identified as an up-regulated gene in both stages, potentially associated with the ripening and color change of prickly ash.

**Fig. 5:**
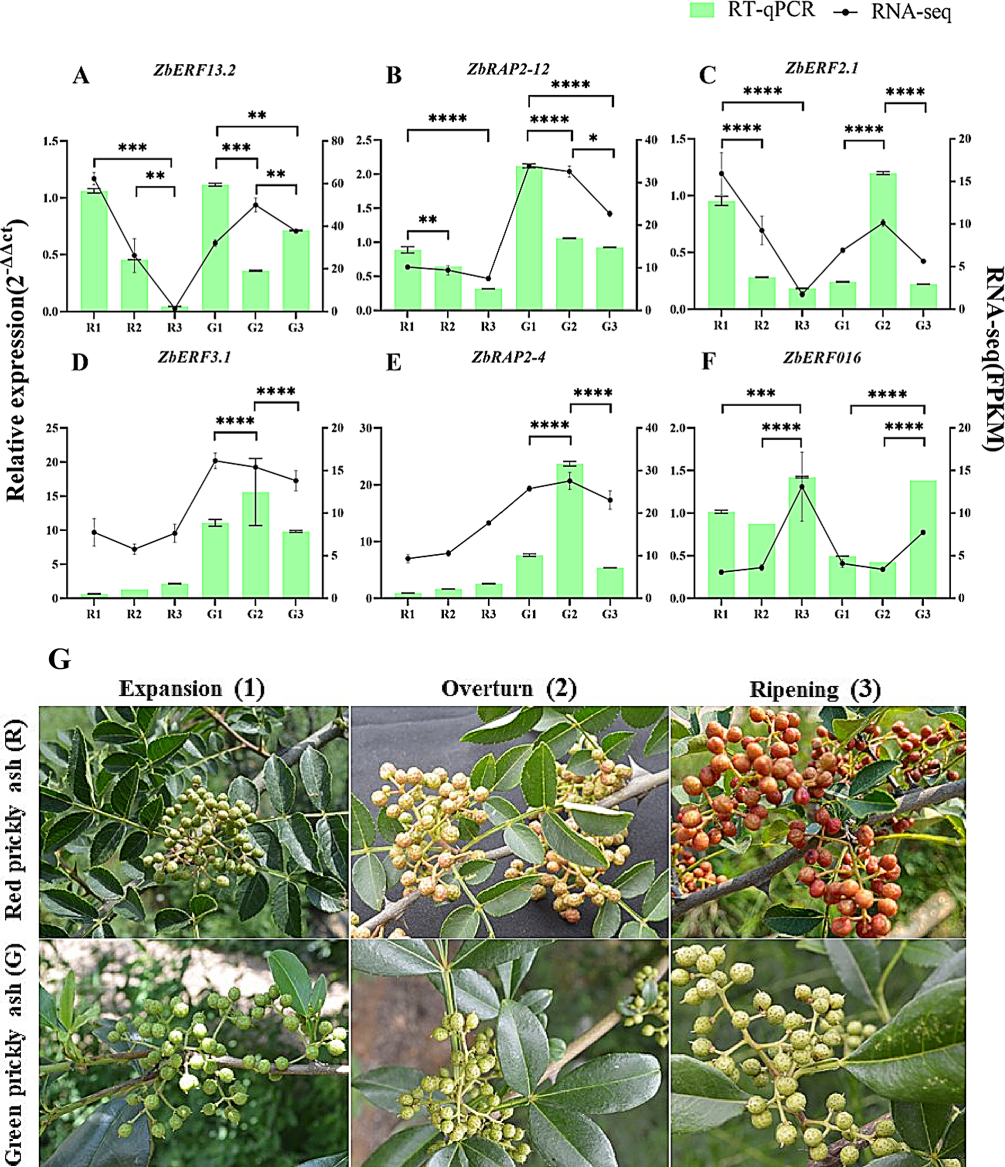
Phenotypes and RT-qPCR expression of genes in red and green prickly ash at different developmental stages. (**A-F**) Expression levels of 6 ZbAP2/ERF genes at different developmental stages. Gene expression levels were calculated relative to the R1 period using the 2^−ΔΔCt^ method. The bar chart shows the mean ± SE of the three biological replicates, with significance levels relative to controls (R1 and G1) indicated by ∗*p* < 0.05; ∗∗*p* < 0.01; ∗∗∗*p* < 0.001. (**G**) Phenotypes of red and green prickly ash fruits at different developmental stages. These three stages respectively correspond to the expansion (R1 and G1) (Fruit enlargement period), coloring (R2 and G2) (Fruit coloring period), and ripening stages (R3 and G3) of Chinese prickly ash fruit

The RT-qPCR results showed that the six ZbAP2/ERF genes exhibited different expression patterns under different periods of fruit development. The expression patterns of the six selected genes were generally consistent with the transcriptome results (Fig. 5). Fit analysis was done comparing RT-qPCR (bar graph) and RNA-seq (line graph) (Fig. 5A-F), and the findings indicated that RT-qPCR confirmed the dependability of transcriptome sequencing. *ZbERF13.2*, *ZbRAP2-12*, and *ZbERF2.1* of red prickly ash (R) were all down-regulated and had significantly different expression levels at each period (Fig. 5A-C); while only *ZbRAP2-12* of green prickly ash(G) showed down-regulation (Fig. 5B). The expression levels of three genes, *ZbERF13.2*, *ZbRAP2-12*, and *ZbERF2.1*, were found to be relatively high in red prickly ash (R) at the expansion stage compared with other developmental periods. The expression levels of *ZbERF3.1* and *ZbRAP2-4* genes were significantly higher in green prickly ash than in red prickly ash, and the high expression was concentrated at the fruit coloration stage (Fig. 5D-E). The expression levels of *ZbERF016* were significantly higher at the ripening stage than at other stages (Fig. 5F). The color and size changes of the fruits of red prickly ash and green prickly ash at different stages of development (Fig. 5G).

### GO and KEGG enrichment analysis

**Fig. 6:**
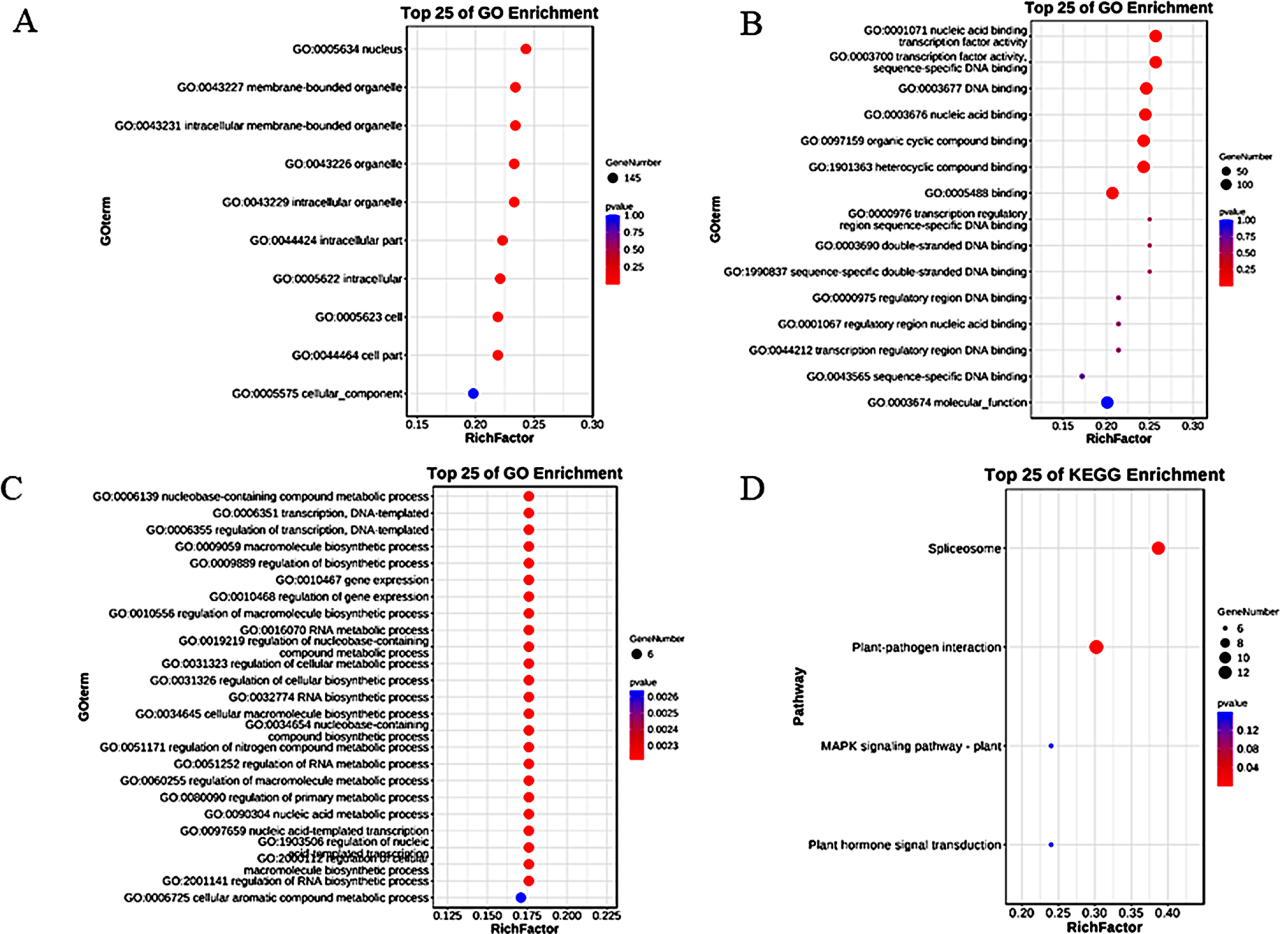
GO and KEGG enrichment. **(A-D)** Enrichment analyses were conducted for cellular components, molecular functions, biological processes, and KEGG pathways. The visualization depicts the size of the circle representing the number of genes, the vertical coordinate representing the GO term or pathway, and the horizontal coordinate representing the p-value, where a smaller value indicates greater significance. Furthermore, only enriched GO terms (i.e., p-value less than 0.05) are displayed

GO database, also known as Gene Ontology (GO), categorizes gene functions into three parts: cellular component (CC), molecular function (MF), and biological process (BP). The GO database enables the identification of the primary functions of genes at the CC, MF, and BP levels. A GO enrichment test was conducted on all genes to unveil the enrichment of gene ontology terms, which revealed statistically significant molecular functions, biological processes, and cellular components (Fig. 6A-C). The top 25 significantly enriched GO terms are displayed, showing that in the cellular component, 9 classes of GO terms were significantly enriched by 145 members. For molecular functions, 7 classes of GO terms were significantly enriched by all member genes, while in biological processes, 24 classes of GO terms were significantly enriched by only 6 genes. Moreover, the KEGG enrichment analysis demonstrated that only two classes of pathways were significantly enriched by 12 genes: Spliceosome and Plant-pathogen interaction (Fig. 6D).

### Predicting interactions between ZbAP2/ERF proteins and other regulatory proteins

**Fig. 7:**
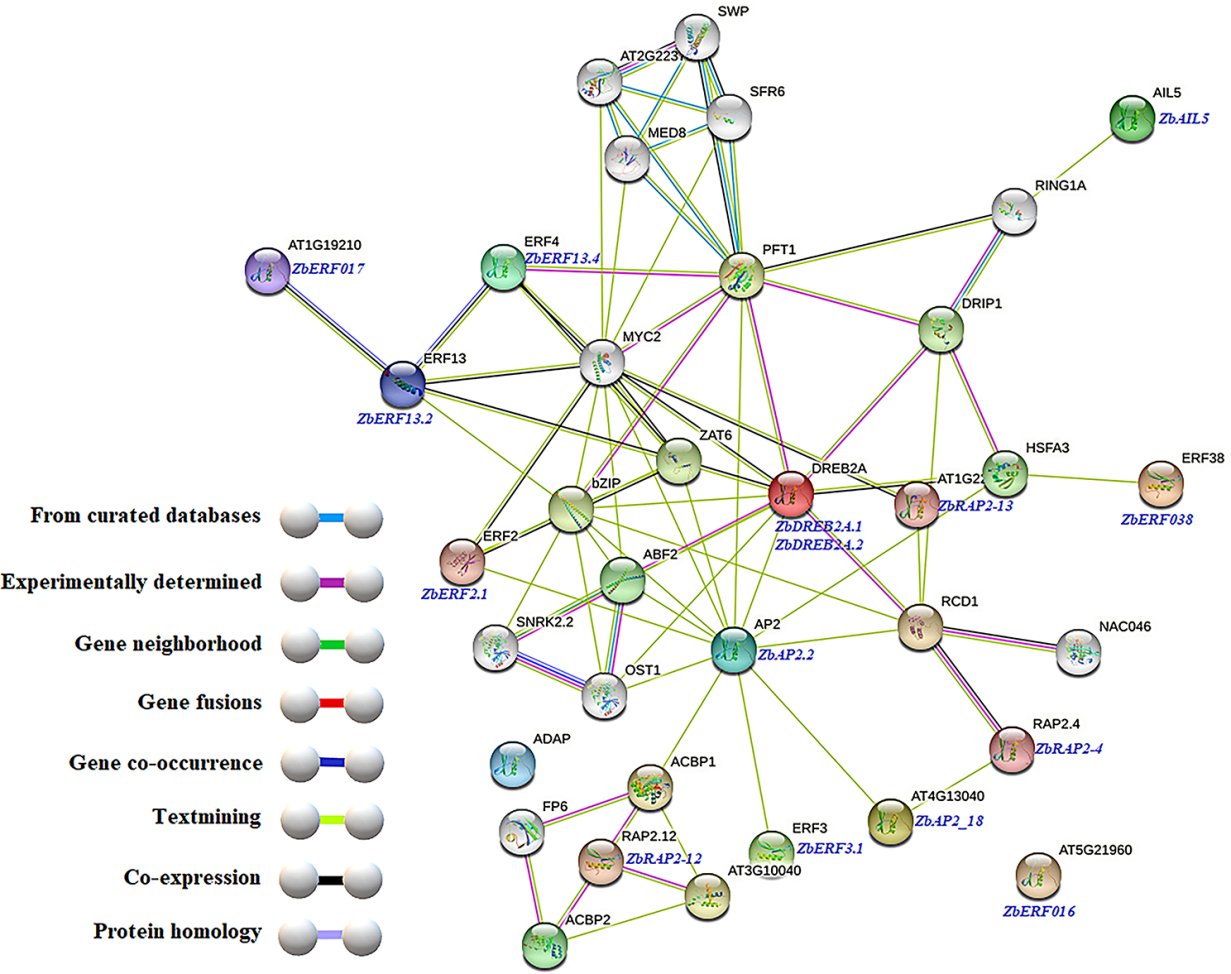
Predicted AP2/ERF protein functional connection network

Interactions between ZbAP2/ERF proteins and other regulatory proteins were predicted with STRING (http://string-db.org/) (Fig. 7). This analysis can reveal interactions that involve direct physical interactions between proteins as well as indirect interactions between functionally related proteins. 15 ZbAP2/ERF genes with robust relative expression levels were selected using transcriptome expression profiling. Using the STRING protein interaction database, functional relationships among these 15 ZbAP2/ERF proteins were predicted using the model plant Arabidopsis thaliana. The results predicted complex interactions with multiple proteins. *ZbDREB2A.1*, *ZbAP2.2*, *ZbERF2.1*, *ZbERF13.2*, *ZbRAP2-12*, *ZbRAP2-4*, *ZbRAP2-12*, and ZbRAP2-13 were predicted to have multiple interactions with other proteins. *ZbAP2.2* was predicted to interact with *ZbDREB2A.1*, *RCD1*, *ACBP1*, *AT3G10040*, *PFT1*, *bZIP*, *ZAT6*, *DRIP1*, *HSFA3*, *ABF2*, and *ACBP2*. Proteins that interact with ZbAP2/ERF proteins may share regulatory pathways that regulate specific physiological responses and plant growth.

## Discussion

AP2/ERF family members have multiple functions in plants, regulating growth and development, biotic and abiotic stress-related transcriptional processes, and affecting fruit ripening, seed germination, and mosaic development. These transcription factors respond positively to invasion by immune pathogens, heat and freezing damage, and drought stress [24]. The increasing availability of plant genome sequence information has allowed in-depth studies on the functional and evolutionary relationships of AP2/ERF transcription factors in a variety of plants, including Arabidopsis (*Arabidopsis thaliana*, 147) [1, 4], rice (*Oryza sativa*, 139) [1], maize (*Zea mays*, 214) [25], sweet potato (*Ipomoea batatas* 198) [26], *Saccharum spontaneum* (218) [27], *Cucurbita moschata* (212) [28], and pomegranate (*Punica granatum*, 116) [29]. However, little is known about the AP2/ERF family in Chinese prickly ash. Therefore, we comprehensively analyzed the ZbAP2/ERF gene family in Chinese prickly ash and completed gene family identification, gene structure analysis, motif analysis, gene ontology annotation, and expression pattern analysis at three developmental stages of fruit. Although the genome sizes of the studied plants vary widely, with 125 Mb for Arabidopsis [30], 2.3 Gb for maize [31], and 4 Gb for prickly ash [32], they contain similar numbers of AP2/ERF transcription factor family members, suggesting that the number of AP2/ERF family members may be relatively stable regardless of the size of the plant genome. It is also illustrated above that the ERF subfamily is characterized by the presence of only a single AP2 structural domain, but some members with a single AP2 structural domain have a high degree of homology to the AP2 subfamily, and these members are classified as the AP2 subfamily rather than the ERF subfamily [1].Conserved motifs in transcription factors frequently play a crucial role in gene function. These motifs are often closely related to protein interactions, transcriptional activity, and DNA binding [4, 33]. Like in other studies of these proteins in other plants, many conserved motifs were found. Kizis revealed that the AP2 structural domain contains YRG, a conserved element associated with DNA binding, and RAYD, a conserved element associated with protein-protein interactions [34]. RAYD can form an amphipathic α-helix that is key to the function of the AP2 domain [35]. Previous multiple sequence comparisons identified a conserved YLG element in the AP2 subfamily and highly conserved WLG elements in the other three subfamilies, and the same pattern of conserved motifs has been described in other reports [36, 37]. Future work should study specific motifs outside the AP2 domain, such as the EAR motif and the EDLL motif in B1 and B3, respectively. Previous studies described an EDLL motif in the AP2 family, a potent plant activation domain that can be used for the transcriptional activation of heterologous DNA-binding proteins [38]. The conserved motif analysis in this paper shows that different subfamilies have different motifs, suggesting different functions. Motifs 1 and 2 are included in all families, and are assumed to be conserved motifs in the AP2 domain. Motifs 1, 2, 3, and 4 are present in most family members. There are also unique motifs in subsets of genes, such as motif 9. The phytohormone ethylene is regulated by several factors during fruit development to ripening, including AP2/ERF TFs [39]. In Japanese pear, *PsERF1* plays vital roles in fruit development and ripening and seven ERF family genes (*PsERF1a, PsERF1b, PsERF2a, PsERF2b, PsERF3a, PsERF3b, PsERF12*) show differential expression during fruit development [13]. We found high expression of *ZbERF2.1*, *ZbERF3.2*, and *ZbERF3.3*, which are homologous to *PsERF2* and *PsERF3* and to the Arabidopsis *AtERF2* and *AtERF3* genes. These differentially expressed genes may suggest some directions for further studies of prickly ash fruit development. Our data showed that *ERF13.2* exhibits the highest expression of the identified genes, and belongs to the *ERF B-3* subgroup like the genes mentioned above.

Green and red prickly ash were studied at different developmental stages. Red prickly ash changed color from green to red as it matured, while green prickly ash did not change much (Fig. 5G). The expression level of some genes in red prickly ash was much higher than that in green prickly ash, and some ZbAP2/ERF genes may be involved in regulating secondary metabolism. Modulation of secondary metabolism has been reported in other plants, such as Arabidopsis, where *AtRAP2.2* specifically regulates leaf carotenoid biosynthesis [40] and in *Artemisia annua*, where *AaERF1* positively regulates artemisinin biosynthesis [41].

Future work should explore the contributions of AP2/ERF transcription factors to secondary metabolism in Chinese prickly ash. These genes are also involved in regulation of many other processes, such as response to abiotic stresses. *OsDREB1A* in rice is involved in drought stress [42], *AtDREB2A* in Arabidopsis is involved in salt stress and high-temperature stress [23], and *AtRAP2.2* in Arabidopsis is involved in regulating hypoxia stress [43]. Similarly, some AP2/ERF genes show changes in response to abiotic stress in in Chinese prickly ash. Although different subfamilies have different functions, there may be shared regulatory mechanisms. The ERF family genes *AtERF4* and *AtERF8*, which have been identified in *Arabidopsis thaliana*, are associated with the process of plant senescence. It is important to note that chlorophyll degradation occurs naturally during plant senescence [44]. It has been demonstrated that transcriptional alterations in *CitERF13*, isolated from the fruit of *Citrus sinensis*, a plant of the same family as Chinese prickly ash, are closely associated with the degree of pericarp development. Transient translational overexpression of *CitERF13* in both tobacco and citrus pericarp led to rapid degradation of chlorophyll, resulting in de-greening of the pericarp. It is postulated that members of the same ERF subfamily of genes in Chinese prickly ash fruits may be involved in regulating color changes during fruit development or senescence. In summary, the AP2/ERF family of transcription factors is involved in a wide range of regulatory pathways, including plant growth, organ development, stress response, signal transduction, and metabolite biosynthesis. This characterization of AP2/ERF family members will provide insight into developmental regulatory mechanisms and help guide plant genetic breeding. By identification and transcriptional expression profiling of the AP2/ERF transcription factor family in Chinese prickly ash, we screened ZbAP2/ERF candidate genes related to fruit development. The results of this work provide some guidance for genetic improvement and molecular breeding of prickly ash and give insight into effects of AP2/ERF transcription factors on fruit development and other plant physiological processes.

## Conclusions

A total of 146 genes encoding ZbAP2/ERF proteins with complete AP/ERF domains were identified in this study, and these genes were divided into the AP2, ERF, DREB, and RAV subfamilies, with one Soloist. The ERF subfamily was further divided into groups B1 to B6, and the DREB subfamily was subdivided into groups A1 to A6. The conserved AP2 domain and motif composition were analyzed for the genes and multiple sequence alignment was performed to analyze their constituent elements. The phylogenetic trees of ZbAP2/ERF and AtAP2/ERF indicated close phylogenetic relationships within the same taxa or subgroups between the two species, providing clues to their potential functions. We analyzed the expression patterns at different stages of fruit development (30, 60, and 90 days after flowering) in prickly ash, with several genes exhibiting developmentally-related changes in gene expression. The expression of some genes in fruit development was verified by RT-qPCR, which matched the transcriptome expression profiles, identifying some candidate genes for further study of their expression and function in plant growth and organ development.

### Methods

#### Identification of the AP2/ERF proteins in Chinese prickly ash

AP2/ERF Proteins in Chinese prickly ash were identified by the Pfam database (http://pfam.xfam.org/) [45] according to the AP2 domain number (PF00847). The HMM model of the AP2 domain was used to identify candidate genes, and at least one AP2 domain was required to be present in each protein sequence, with a cutoff value of 0.01. All ZbAP2/ERF family gene sequences were searched for homologous genes using the sequence search tool of the Pfam database, and the ZbAP2/ERF family genes were named according to the homologous genes with the highest matches, and the sources of the relevant homologous genes are shown in Supplementary Table 1. Use the CD-hit online tool (http://weizhong-lab.ucsd.edu/cdhit_suite/) to remove repetitive sequences in the AP2/ERF transcription factor family. The putative AP2/ERF proteins were further confirmed using BLASTP and the Conserved Domain Database (CDD, http://www.ncbi.nlm.nih.gov/cdd/) [33]. Protein physicochemical parameters, including the number of amino acids, molecular weight, and PI value, were predicted using ExPasy website tools (http://web.expasy.org/protparam/) [46].

#### Multiple sequence alignment and phylogenetic analysis of ZbAP2/ERF TFs

The phylogenetic tree of the AP2/ERF protein sequences of *Zanthoxylum bungeanum* and *Arabidopsis thaliana* AP2/ERF protein sequences was constructed using MEGA-X software [47]. The neighbor-joining method was used with default parameters (the model contains a Poisson correction). The AP2/ERF protein sequences of Arabidopsis thaliana were obtained by downloading from the TAIR website (http://www.arabidopsis.org/). The phylogenetic tree file generated by MEGA software was uploaded to the online website (https://itol.embl.de/) [48] for further processing of the phylogenetic tree. Multiple sequence alignment of AP2/ERF protein sequences was achieved using ClustalW and default parameters. The alignment results and groupings were then analyzed using ESPript3.x (https://espript.ibcp.fr/ESPript/ESPript/) [49].

#### Conserved motif search and domain visualization of ZbAP2/ERF TFs

The ZbAP2/ERF protein sequences of each subfamily were submitted to the online website MEME (http://meme-suite.org/tools/meme) [50] to identify motifs. The classical pattern was selected as the base motif discovery pattern, and the number of motifs selected for discovery was 10. The visualization of the motifs was achieved using TBtools software according to the files generated by the MEME website [51]. The ZbAP2/ERF sequences were uploaded to the NCBI website to search for domains using the Batch CD-search tool and then visualized using TBtools software.

#### ZbAP2/ERF expression pattern analysis and prediction

Clustering heatmap analysis was performed and the results were visualized using TBtools software. Enrichment analyses of differentially expressed genes (DEGs) were conducted using the OmicShare tools, which is a freely accessible online platform for data analysis (http://www.omicshare.com/tools) Used DEGseq2 to analyze differentially expressed genes. Significance levels were based on two-way ANOVA and Tukey’s test by using GraphPad Prism 9 software.

#### Plant materials

Fruit samples of *Zanthoxylum bungeanum* (red prickly ash) and *Zanthoxylum armatum* (green prickly ash) were picked at the prickly ash demonstration plantation of Northwest Agriculture and Forestry University (N33°59′6.55′′E106°39′29.38′′). Red (R) and green (G) fruit samples were collected at three different developmental stages: 30 days after flowering (R1 and G1), 60 days after flowering (R2 and G2), and 90 days after flowering (R3 and G3). These three stages respectively correspond to the expansion (Fruit enlargement period), coloring (Fruit coloring period), and ripening stages of Chinese prickly ash fruit. Three biological replicates were collected for each developmental stage and the harvested fruit samples were stored in liquid nitrogen and then transferred to an ultra-low temperature freezer at -80 °C.

#### RNA extraction, sequencing and RT-qPCR detection and data analysis

TaKaRa MiniBEST Plant RNA Extraction Kit (TaKaRa, Beijing, China) was used to extract total RNA. A NanoDrop 2000 spectrophotometer (Thermo Scientific, Pittsburgh, PA, USA) was used to evaluate the purity, concentration, and integrity of RNA samples. High-quality RNA was used to construct cDNA libraries, and the resulting 150-bp paired-end reads were sequenced on the Illumina NovaSeq 6000 platform. High-quality reads were obtained by mapping clean reads to assembled transcripts and calculating gene expression levels in fragments per kilobase per million (FPKM) by trimming sequencing bridges and removing low-quality reads from raw sequencing data [22].

The ToloScript RT EasyMix Kit (TOLOBIO) was used to synthesize first strand cDNA. To investigate gene expression patterns, 6 highly expressed ZbAP2/ERF representative genes were selected. Primer Premier 5.0 was used to design the RT-qPCR primers. Supplementary Table 1 lists all primers used for RT-qPCR. To normalize the RT-qPCR results, UBQ was used as a reference gene [52]. RT-qPCR experiments were performed using the CFX Connect PCR Detection System (Bio-Rad, United States) and Q3 SYBR qPCR Master Mix (TOLOBIO). For all samples, three biological replicates and three technical replicates of each biological replicate were used. RT-qPCR protocols were: initial denaturation at 95 °C for 2 min, denaturation at 95 °C for 10 s, and denaturation at 60 °C for 30 s. A total of 40 cycles were run using the instrument’s default lysis curve program. The 2^−ΔΔCt^ method was used to calculate gene expression levels relative to the R1 period. Statistical analysis and graphing were performed with GraphPad Prism 9.5.0 and all data analyzed by one-way ANOVA (95% confidence range). The Tukey multiple comparison test was used to test for differences.

### Electronic supplementary material

Below is the link to the electronic supplementary material.

Supplementary Material 1

Supplementary Material 2

## Data Availability

The transcriptome data of Chinese prickly ash has been submitted to the NCBI database (https://www.ncbi.nlm.nih.gov/) with the accession number (PRJNA756714). All data generated or analyzed during this study are included in this article and its supplementary information files. The datasets generated and analyzed during the current study are available from the corresponding author on reasonable request. The data websites used in this study are as follows: the Tair database (https://www.arabidopsis.org/index.jsp), the Pfam database (http://pfam.xfam.org/), the Plant Transcription Factor Database (http://planttfdb.gao-lab.org/).

## Abbreviations

TF: Transcription Factor
FPKM: Fragments Per Kilobase Of Transcript Per Million Mapped Fragments
MW: Molecular Weight
PI: Isoelectric Point
aa: amino acid
W: Tryptophan
L: Leucine
G: Glycine
Y: Tyrosine
R: Arginine
I: Isoleucine
V: Valine
S: Serine
C: Cysteine
A: Alanine
N: Asparagine
T: Threonine
E: Glutamic acid
D: Aspartic acid
RCD1: Radical-induced cell death 1
ACBP1: Acyl-CoA-Binding Domain-Containing Protein 1
AT3G10040: Sequence-specific DNA binding transcription factors
PFT1: Phytochrome and Flowering Time Regulatory Protein
bZIP: Basic leucine-Zipper 8
ZAT6: Zinc finger of Arabidopsis Thaliana 6
DRIP1: E3 ubiquitin protein ligase
HSFA3: Arabidopsis thaliana heat shock transcription factor a3
ABF2: Abscisic acid responsive elements-binding protein 2
ACBP2: Acyl-CoA-binding domain-containing protein 2
