# Supplementary material for: Genome-wide analysis of AP2/ERF transcription factors that regulate fruit development of Chinese prickly ash
Source: BMC Plant Biol. 2024 Jun 15;24:565. doi: 10.1186/s12870-024-05244-9 (PMC11179286; doi:10.1186/s12870-024-05244-9)
Supplement: Supplementary file 1 — Supplementary Material 1 [file 12870_2024_5244_MOESM1_ESM.docx]

Supplementary Material

Genome-wide analysis of AP2/ERF transcription factors that regulate fruit development of Chinese prickly ash

Lei Ma^1,2, #^,Qianqian Shi^1, #^, Qin Ma^1,2^，Xiaona Wang^1,2^，Xin Chen^1,2^, Peilin Han^1,2^, Yingli Luo^1,2^, Haichao Hu^1,2^, Xitong Fei^1,2*^ and Anzhi Wei^1,2*^

^1^College of Forestry, Northwest Agriculture and Forestry University, Yangling, Xianyang 712100, China

^2^Research Centre for Engineering and Technology of Zanthoxylum State Forestry Administration, Yangling, Xianyang 712100, China

^#^These authors contributed equally to this work.

*** Correspondence:**

Xitong Fei

feixitong@nwafu.edu.cn

Anzhi Wei

weianzhi@nwafu.edu.cn

# Supplementary Figure


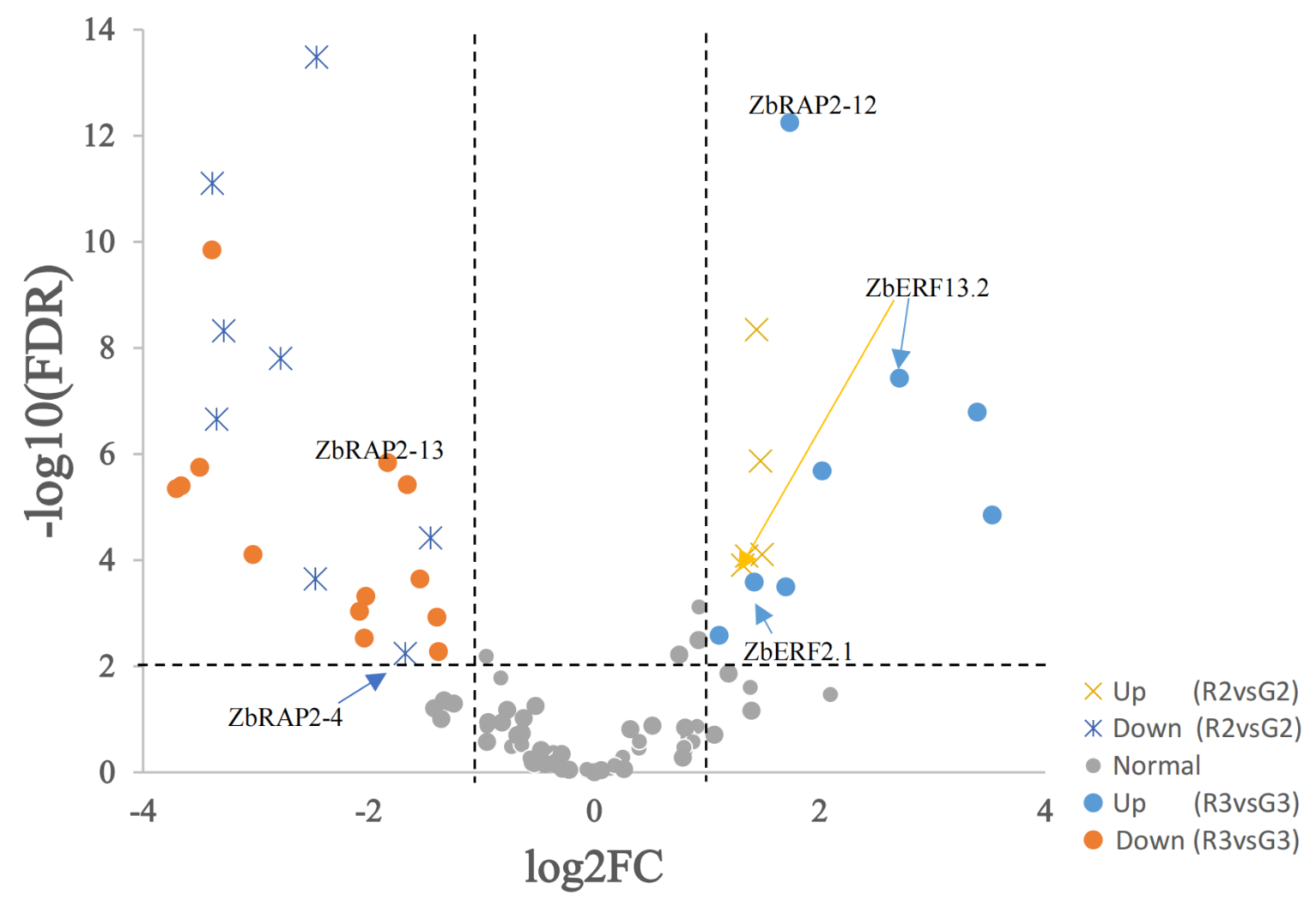


## Supplementary Figure 1 Analysis of differentially expressed genes during the ripening period of green and red Chinese prickly ash. In the visualization, blue circles indicate up-regulated genes at maturity (log2FC>1, FDR<0.01), orange circles represent down-regulated genes at maturity (log2FC<-1, FDR<0.01), yellow × represents up-regulated genes at the coloring stage (log2FC>1, FDR<0.01), blue × represents down-regulated genes (log2FC<-1, FDR<0.01), and grey denotes genes that are not statistically significant.

##
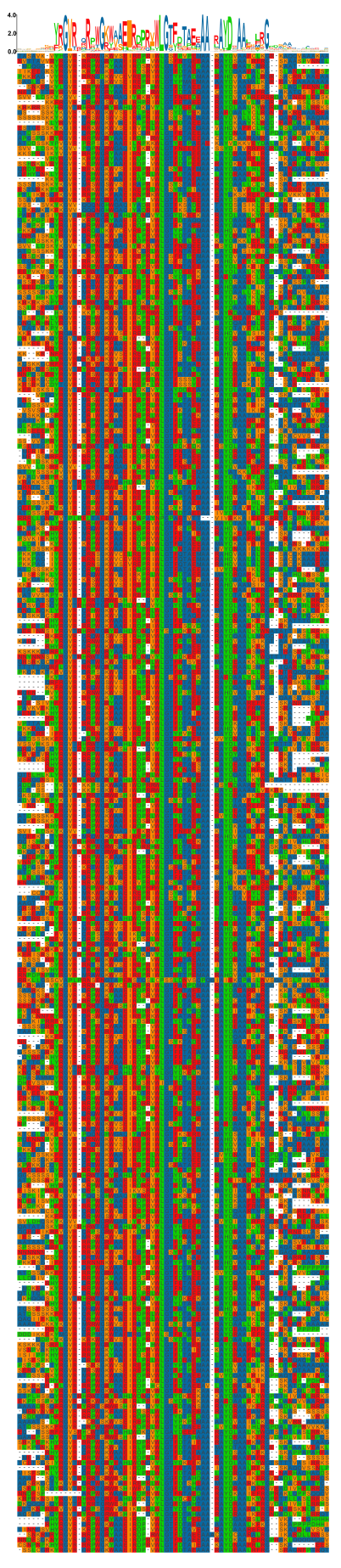


## Supplementary Figure 2 AtAP2/ERF and ZbAP2/ERF Gene Multiplex Sequence Alignment Results.

#
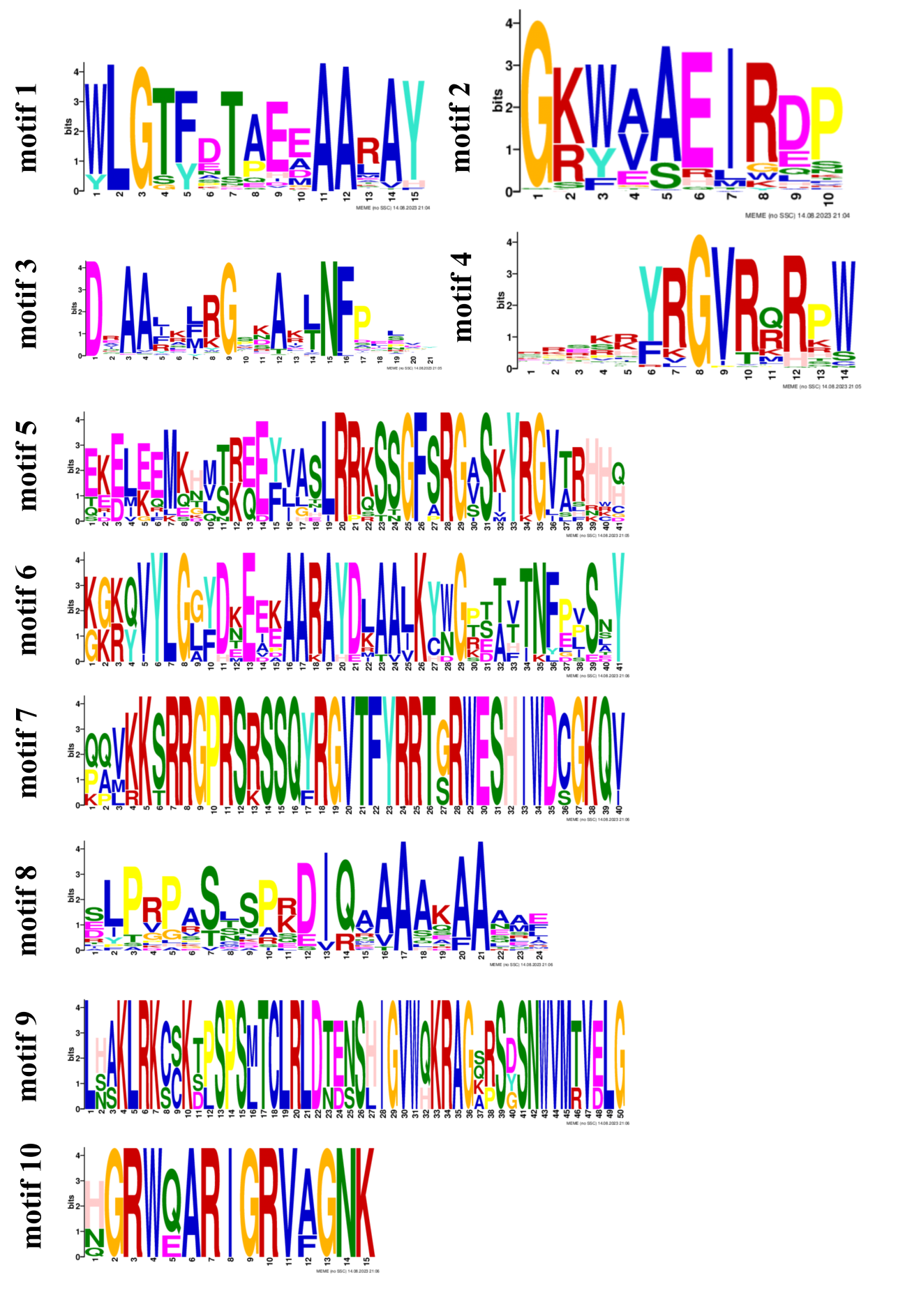


## Supplementary Figure 3 Logos of the 10 discovered motifs.
